# Parinaud Oculoglandular Syndrome Among Female Adults in Malaysia: A Case Series

**DOI:** 10.7759/cureus.67066

**Published:** 2024-08-17

**Authors:** Nur Nadia Abd Rahim, Mazita Ami, Zurina Zainal Abidin

**Affiliations:** 1 Otolaryngology - Head and Neck Surgery, KPJ Healthcare University, Nilai, MYS; 2 Ophthalmology and Oculoplastic Surgery, KPJ Klang Specialist Hospital, Klang, MYS

**Keywords:** general practice, family medicine, pets, bacteria, zoonotic, bartonella henselae, lymphadenopathy, ophthalmology, cat scratch disease, parinaud oculoglandular syndrome

## Abstract

Parinaud oculoglandular syndrome (POGS), is typically rare and often presented as a unilateral ocular inflammation accompanied by ipsilateral lymphadenopathy. POGS is an atypical manifestation of cat scratch disease (CSD) caused by *Bartonella henselae* (BH). Diagnosis of POGS poses a challenge due to its rarity and the array of potential etiologies including infections from fleas, ticks, and various microorganisms. This case series details three cases of CSD attributed to POGS, highlighting the diagnostic challenges faced in the absence of the gold standard diagnostic method, which is the polymerase chain reaction (PCR) DNA test for BH. The cases encompass a set of presentations including granulomatous inflammation and lymphadenopathy, managed effectively with antibiotics and non-pharmacological interventions such as flea control in domestic felines and hygiene measures post-feline inflicted injury. These cases highlight the necessity for heightened clinical suspicion, especially in patients with a history of feline contact, and appeal for further research to refine diagnostic criteria for more accurate and practical detection of CSD particularly for the atypical manifestations. This will be especially beneficial in areas where the more invasive lesion biopsy or gold standard PCR DNA test for BH are not available so accurate management can be instituted immediately in cases where there is multisystemic involvement.

## Introduction

Henri Parinaud, an ophthalmologist from France first described the oculoglandular syndrome in 1889, which has come to be known as the ocular bartonellosis, describing three patients with fever, regional lymphadenopathy (preauricular and submandibular lymph nodes), and a follicular conjunctivitis [1]. Parinaud oculoglandular syndrome (POGS) patients usually complain of unilateral eye redness, foreign body sensation, and excessive tearing. Diagnosing POGS is more difficult for physicians as it is considered a rare ophthalmological presentation of cat scratch disease (CSD). Furthermore, various case reports and a review describe multiple causes of POGS, which include fleas, ticks, tuberculosis, and Epstein-Barr virus [2]. It has also been reported that there was a mix of associated symptoms and various onsets for each symptom for each patient. Here we report three cases of POGS caused by CSD (*Bartonella henselae* or BH) with the challenges in confirming the diagnosis using a set of criteria suggested by Margileth AM without the availability of the gold standard testing which is polymerase chain reaction (PCR) DNA for BH [3,4].

## Case presentation

Case 1

**Figure 1:**
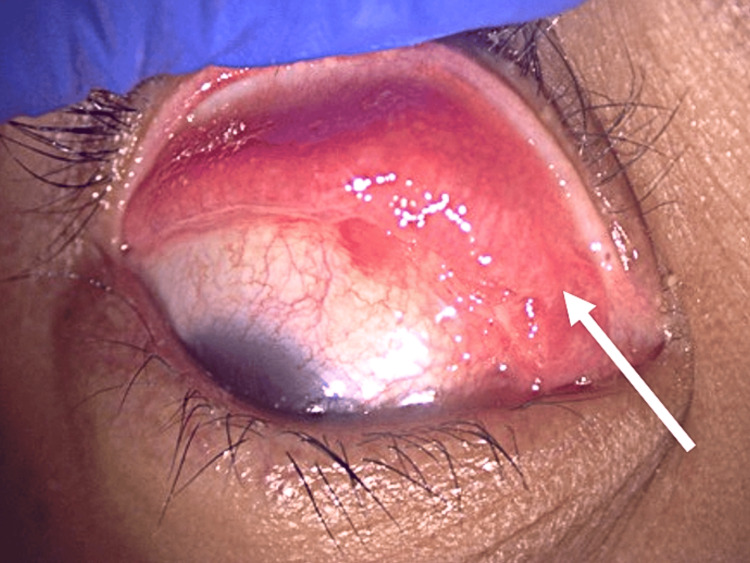
Case 1 showing right upper eyelid mass and follicles (white arrow)

**Figure 2:**
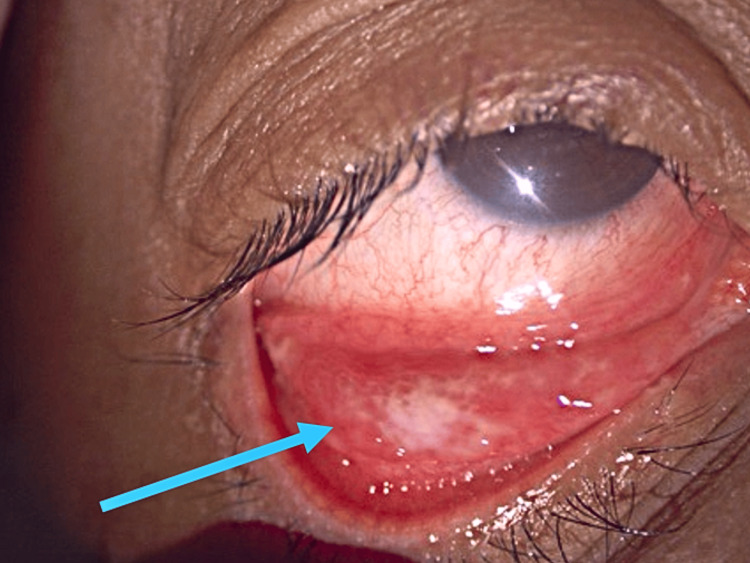
Case 1 showing right lower fornix follicles biopsy site (blue arrow)

This 44-year-old female cat rescuer with no previous medical illness presented with a painless right eye swelling with associated watery discharge for the past one month. There was no itchiness in her right eye and no nasal symptoms like nasal discharge or nasal stuffiness, but there was a right-sided cheek swelling. She had frequent contact with a stray cat on sporotrichosis treatment. Physical examination showed erythematous right upper and lower eyelids with the presence of follicles (Figures 1, 2).

Fundus, visual acuity, retina, anterior and posterior compartments, and intraocular pressure (IOP) (14 mmHg; mean IOP in Asian females in all age groups is 14.9 mmHg) were unremarkable. There was also a whitish patch on her right upper nasal area with a right pre-auricular mobile, non-tender lymph node palpable measuring about 2 x 1.5 cm. Other systemic examinations were unremarkable with normal full blood count readings and her erythrocyte sedimentation rate (ESR) was normal at 7 mm/h (normal range: <20 mm/hour; age and gender adjusted). Her sexually transmitted infection screening laboratory workup was negative (*Neisseria gonorrhoeae* DNA, *Chlamydia trachomatis*, *Trichomonas vaginalis*, *Mycoplasma hominis*, *Mycoplasma genitalium*, *Ureaplasma urealyticum*, and Ureaplasma parvum) thus excluding it as the possible cause of the eye symptoms. The PCR tests for *Mycobacterium tuberculosis* (MTB) and non-*Mycobacterium tuberculosis *(NTM) were negative as well. A computed tomography (CT) scan of the paranasal sinuses (PNS)/head revealed a 1.0 x 0.9 right intraparotid lymph node with a 2.2 cm right submandibular lymph node. BH serology was equivocal and her right lower fornix follicles biopsy showed granulomatous inflammation with a few suppurative granulomas. The Warthin-Starry silver stain to detect BH was negative for this patient. The biopsy was negative for fungal and acid-fast tuberculous bacilli. She was treated with azithromycin eye drops twice daily (BD), T. cefuroxime 250 mg BD for two weeks, T. fluconazole 150 mg weekly for four weeks, and T. doxycycline 100 mg BD for two months. This patient achieved complete resolution of her symptoms at the end of the treatment.

Case 2

**Figure 3:**
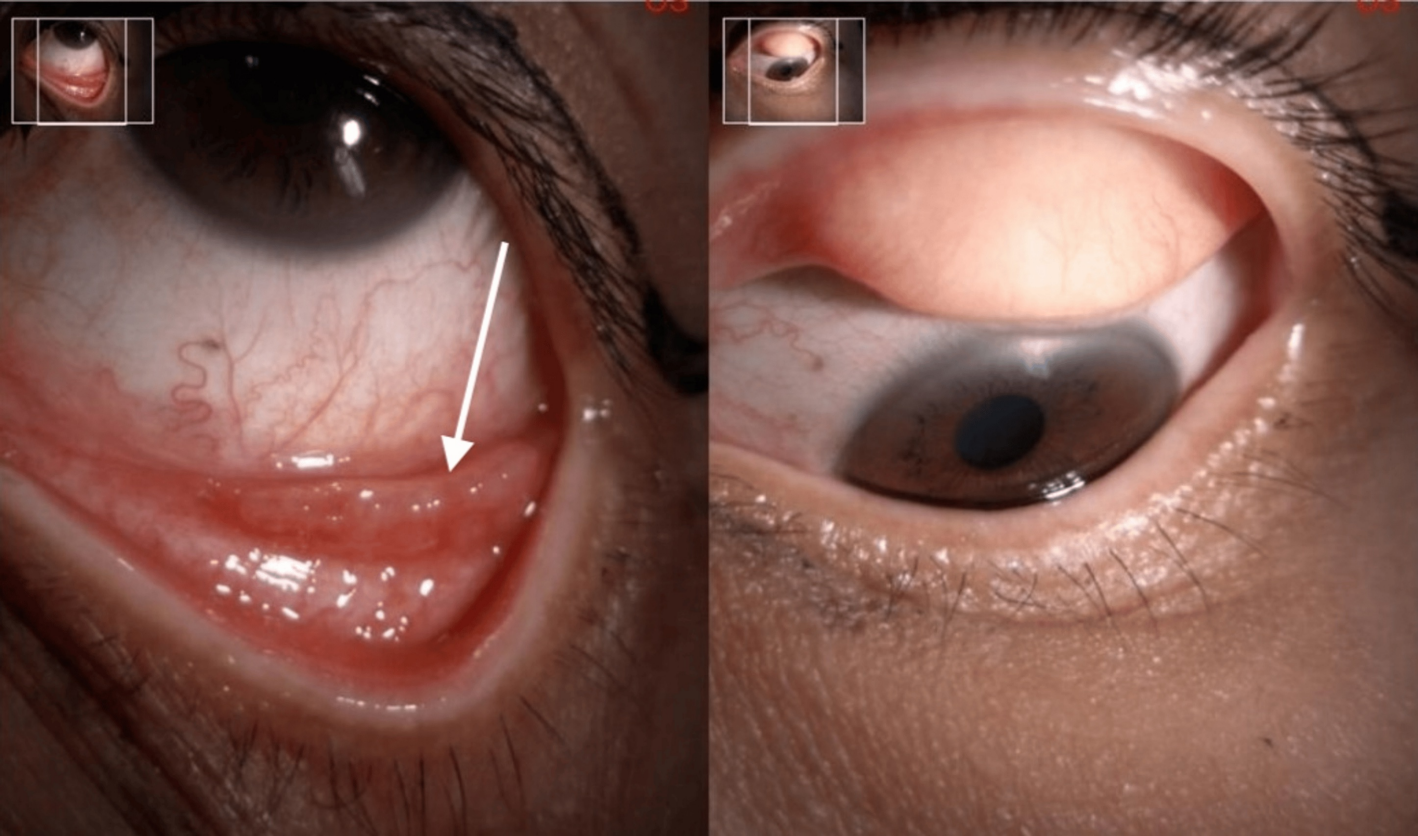
Case 2 showed a granulomatous nodule on her left lower tarsal conjunctiva (white arrow)

A 50-year-old female cat owner with no previous medical illness presented with left eye redness for the past one month associated with an ipsilateral left cheek swelling. She reported having a short episode of fever prior to the onset of the left cheek swelling. She was treated with a one-week course of oral antibiotics prior to her presentation to the ophthalmology clinic. Despite the improvement of her eye symptoms, her left cheek swelling remained. There were no nasal symptoms reported. Physical examination revealed bilateral blepharitis with the left eye appearing injected. There was a follicular lesion with papillae at the fornix of the left lower eyelid (Figure 3).

The ocular examination was unremarkable with a normal IOP of 14 mmHg. She also had multiple left pre-auricular lymph nodes palpable with a single left level II lymph node. Her lymph nodes are non-tender and mobile. Her full blood count was normal except her ESR and her C-reactive protein (CRP) were raised at 51 mm/hr (normal range: <20 mm/hour; age and gender adjusted) and 11.18 mg/dL (normal range: < 5mg/dL, no age and sex adjustment) respectively. All her sexually transmitted infection screenings including PCR for MTB and NTM were negative. The BH serology (IgG and IgM) were positive and the left lower tarsal conjunctiva biopsy result showed left chronic granulomatous follicular conjunctivitis with negative results for fungal and acid-fast tuberculous bacilli. The Warthin-Starry silver stain did not detect BH in this patient. Her CT scan PNS showed left intraparotid and pre-auricular nodes with left level I and II cervical lymphadenitis. She was treated with an azithromycin eye drop BD and intravenous broad-spectrum antibiotics (ceftriaxone and azithromycin for three days) before started on T. doxycycline 100 mg BD for two months. This patient, clinically recovered completely from POGS.

Case 3

**Figure 4:**
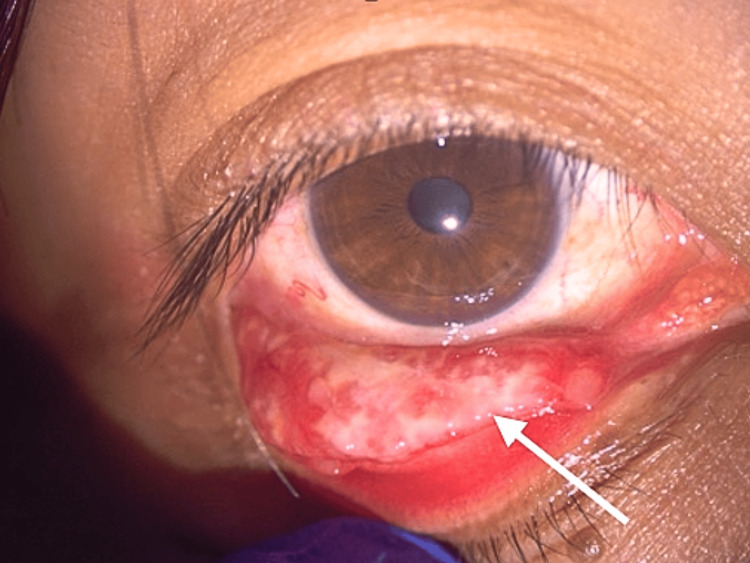
Case 3 right lower eyelid elongated mass at fornix (white arrow indicating biopsy site)

A 42-year-old lady with no previous medical illness presented with a history of cat bites from a sporotrichosis-infected cat presented with a right lower eyelid swelling with an ipsilateral pre-auricular non-tender lymphadenopathy for three weeks. She was treated with oral azithromycin for three days but showed no signs of improvement. There were no other eye or nasal symptoms. On examination, it was found that her right eye's lower lid is edematous, with the presence of an elongated mass with follicles and a suppurative membrane over the inferior fornix (Figure 4).

The ocular examination revealed no significant findings with a normal IOP of 14 mmHg. Her full blood count results including CRP were normal, as well as all the sexually transmitted infection screenings came back negative. PCR for MTB, NTM, and *Chlamydia *together with BH serology were negative. The right lower fornix elongated mass biopsy result showed necrotizing granulomata seen but no presence of caseation. The Warthin-Starry silver stain for detecting BH yielded negative results in this patient. The fungal and acid-fast tuberculous bacilli for the specimen were negative as well. No imaging studies were done for this patient. She was started on eye drop azithromycin eye drop BD, T. doxycycline 100 mg BD for two months, and T. fluconazole 200 mg stat and then 150 mg weekly for four weeks. This patient reported complete remission of her symptoms.

## Discussion

The first diagnosis of CSD was done by Robert Debre, a French physician in 1931 when a 10-year-old boy presented with lymphadenopathy, suspicious of tuberculosis [5]. It was not until 50 years later that the team led by pathologist, Douglas J. Wear identified a gram-negative, rod-shaped, non-motile, facultative intracellular zoonotic bacillus; BH when examining a lymph node biopsy specimen from an 11-year-old girl [6]. The prevalence of BH is up to 16.6% in cats in Asia and 15.3% worldwide as discovered by a study [7] and between 92.4% to 99.1% of cases of CSD reported having a history of feline contact [8,9].

Most CSD infection symptoms are usually self-limiting and mild. The common symptoms include constitutional symptoms, i.e., generalized aching, malaise, and anorexia while the atypical manifestation includes granulomatous POGS, optic nerve edema, subretinal fluid/exudates, uveitis, erythema nodosum, encephalopathy, osteolytic lesion, thrombocytopenic purpura and erythema marginatum accounts. These atypical symptoms account for 5% of the total cases [9]. Of these, POGS was the most common, constituting 80% of atypical cases. Diagnosis is complicated by the delayed onset of certain symptoms, like lymphadenopathy, which may emerge up to three weeks post-infection, as was observed in one case similar to the second case in this series [10]. The palpebral conjunctiva is often involved, presenting with soft granulation at the inoculation site [9].

The challenge lies in diagnosing CSD due to the atypical symptoms with variable onset, coupled with the limited resources in Malaysia, as it is a complex process that involves integrating epidemiological, histological, and bacteriological criteria. In typical presentations, three out of four criteria are needed as compared to all four criteria for atypical presentations. The four criteria suggested include cat or flea contact with or without a scratch mark or a regional inoculation lesion (skin papule, eye granuloma, mucous membrane); negative laboratory findings for other causes of lymphadenopathy or a positive PCR for BH or a liver/spleen abscess on CT scan; a positive enzyme indirect fluorescent antibody (IFA) or IFA assay serology tests; and a biopsy result of the lesion which shows granulomatous inflammation compatible with CSD or a positive Warthin-Starry silver stain [3]. Hence, for a positive serological assay result in a patient with prior feline contact accompanied by the presence of CSD granuloma and negative results for other causes of lymphadenopathy, it is recommended that the physician perform a lymph node/clinical lesion biopsy for cases suspected of CSD [4].

At present, the identification of BH DNA in lymph nodes or clinical lesions using molecular techniques is considered the gold standard for diagnosis because of the superior sensitivity and specificity this method offers compared to others [4]. Nevertheless, the utilization of this method of diagnosis is severely limited due to the invasive nature of the sampling methods such as incisional or excisional biopsy of the lesion or lymph nodes [11], which may require general anesthesia, especially in the pediatric population. As of the time this article is written, PCR for BH from tissue biopsy is not readily available in Malaysia due to the high cost it incurs.

POGS secondary to BH has been successfully managed using oral antibiotics and corticosteroids, depending on the current condition of the eye. A local retrospective study on the clinical spectrum of ocular bartonellosis showed that the occurrence of POGS was 23.1% in the population studied with an average treatment duration of two weeks with antimicrobial, the most commonly used being doxycycline [12]. The clinician must observe the progress of the symptoms as even with a positive IFA, and the commencement of oral antibiotics, the patient might not improve clinically. A local case reported antibiotic treatment failure (azithromycin and doxycycline) in a patient, and despite the negative tuberculosis test results, an anti-tuberculous regimen was started [13]. Fortunately, the patient showed an improvement clinically and the diagnosis was revised to POGS secondary to conjunctiva tuberculosis, in view of the geographical factor of this case, situated in a tuberculosis-endemic country [13].

The most common antibiotics recommended for mild to moderate CSD are a five-day course of azithromycin with other antibiotics such as rifampicin, doxycycline, ciprofloxacin, gentamycin, and trimethoprim-sulfamethoxazole. The duration of treatment is between two to four weeks for immunocompetent patients, while for immunocompromised patients it may last for up to four months [14]. In this case series, the antibiotic of choice was doxycycline which was given for a course of eight weeks due to the diagnosis of POGS. None of these patients were pregnant and they had no existing gastrointestinal issues. All three patients recovered fully and had no further complications.

Additional non-pharmacological strategies that may be suggested for managing POGS secondary to BH incorporate a multifaceted approach. Patients are counseled to implement comprehensive flea control measures for domestic felines and to maintain these pets within indoor environments to curtail zoonotic transmission. Furthermore, it is crucial to reduce human contact, particularly among susceptible demographics including pediatric, geriatric, and immunodeficient populations, with stray animals or pets of undetermined health status. Immediate and thorough disinfection of any feline-inflicted wounds with warm, soapy water is advised, coupled with a precaution to prevent animals from licking the injuries. Finally, routine screening and treatment for *Bartonella* in pets are recommended, particularly for those individuals who are at an increased risk of developing serious infections [15].

The precise population of stray dogs and cats in Malaysia is not currently established, but estimates suggest that the populations might be as high as approximately 6 million for dogs and 5 million for cats. These figures significantly surpass the population of owned dogs and cats within the nation [16]. The trend of adopting and treating pets more like family members, known as pet humanization, has notably increased in Malaysia, placing it in the top 10 countries where this phenomenon has spiked, especially in the year 2020 compared to the past five years [17]. This shift in pet ownership and trend must be accompanied by increased awareness of possible more widespread zoonotic infections in the local population.

## Conclusions

It is important to have a high index of suspicion for patients who present with evidence of lymphadenopathies and a history of feline contact given the increased feline contact in the local population. The varied clinical manifestations and the absence of widely accessible, definitive polymerase chain reaction DNA testing for *Bartonella henselae* strengthen the need for clinicians to maintain vigilance and a high degree of clinical suspicion, particularly in patients with a history of exposure to felines. The current management of the prescription of antibiotics with or without corticosteroids, and non-pharmacological measures has proven to be successful. The experiences detailed in this series advocate for the formulation of localized guidelines and diagnostic criteria that can be adapted to the resource capabilities of different regions, potentially improving the prognosis and management outcomes for Parinaud oculoglandular syndrome.
